# Inside the membrane: a closer look using elastic scattering techniques and friends

**DOI:** 10.1007/s00249-025-01761-z

**Published:** 2025-06-26

**Authors:** Michael Kaltenegger, Enrico F. Semeraro, Georg Pabst

**Affiliations:** 1 Biophysics, Institute of Molecular Biosciences, University of Graz, NAWI Graz, Humboldtstr. 50/III, 8010 Graz, Austria; 2 Field of Excellence, BioHealth, University of Graz, Universitätspl. 3, 8010 Graz, Austria; 3 Institute of Molecular Biosciences, University of Graz, Humboldtstr. 50, 8010 Graz, Austria

**Keywords:** Bilayer, Data modeling, Lipids, Proteins

## Abstract

Biological membranes are highly dynamic and adaptive interfaces that define cellular compartments, posing significant challenges for detailed characterization. Among the diverse range of experimental and computational techniques, small-angle scattering emerges as a label-free, non-invasive method capable of probing membrane structures across length scales from micrometers to subnanometers. By exploiting the complementary contrasts of X-ray and neutron scattering, combined with advanced optimization algorithms, this approach has provided unique insights into membranes with well-defined lipid and protein architectures. In this review, we highlight recent studies from the Pabst Lab, including investigations of lipid domains, asymmetric lipid membranes, and intrinsic lipid curvature. Furthermore, we explore the functional implications of these findings, such as the activity of an integral membrane enzyme and the effects of antimicrobial peptides in live cells. These examples underscore the versatility of small-angle scattering techniques in elucidating membrane functions, offering valuable perspectives for understanding cellular processes and advancing pharmaceutical applications.

## Introduction

All physiological processes occur within or near membranes. A fundamental understanding of membrane function is therefore essential for accessing and potentially influencing these processes, whether in healthcare, medical treatment, or biotechnological applications. Biological membranes serve not only as interfaces between cellular compartments but also as crossroads of various scientific disciplines, including molecular biology, biochemistry, soft matter physics, and physical chemistry. The application of principles from these latter disciplines provides an avenue for deconstructing the dynamic and compositional complexity of cell membranes into components that can be quantitatively analyzed using a broad range of experimental and computational techniques.

Among these techniques, small-angle scattering—utilizing either X-rays (SAXS) or neutrons (SANS)—occupies a distinctive and often underappreciated position. Pioneered by scientists such as Guiner and Fournet (1955) and Glatter and Kratky (1982), small-angle scattering is a highly versatile method. It provides ensemble-averaged structural information, including statistical insights into fluctuations ranging from single molecules to collective dynamics. For membranes, this capability allows small-angle scattering to bridge continuum properties, such as those described by the classical Helfrich model of membrane elasticity (Helfrich 1973), with molecular-scale features, such as hydrocarbon chain lengths and the lateral area per lipid.

**Fig. 1:**
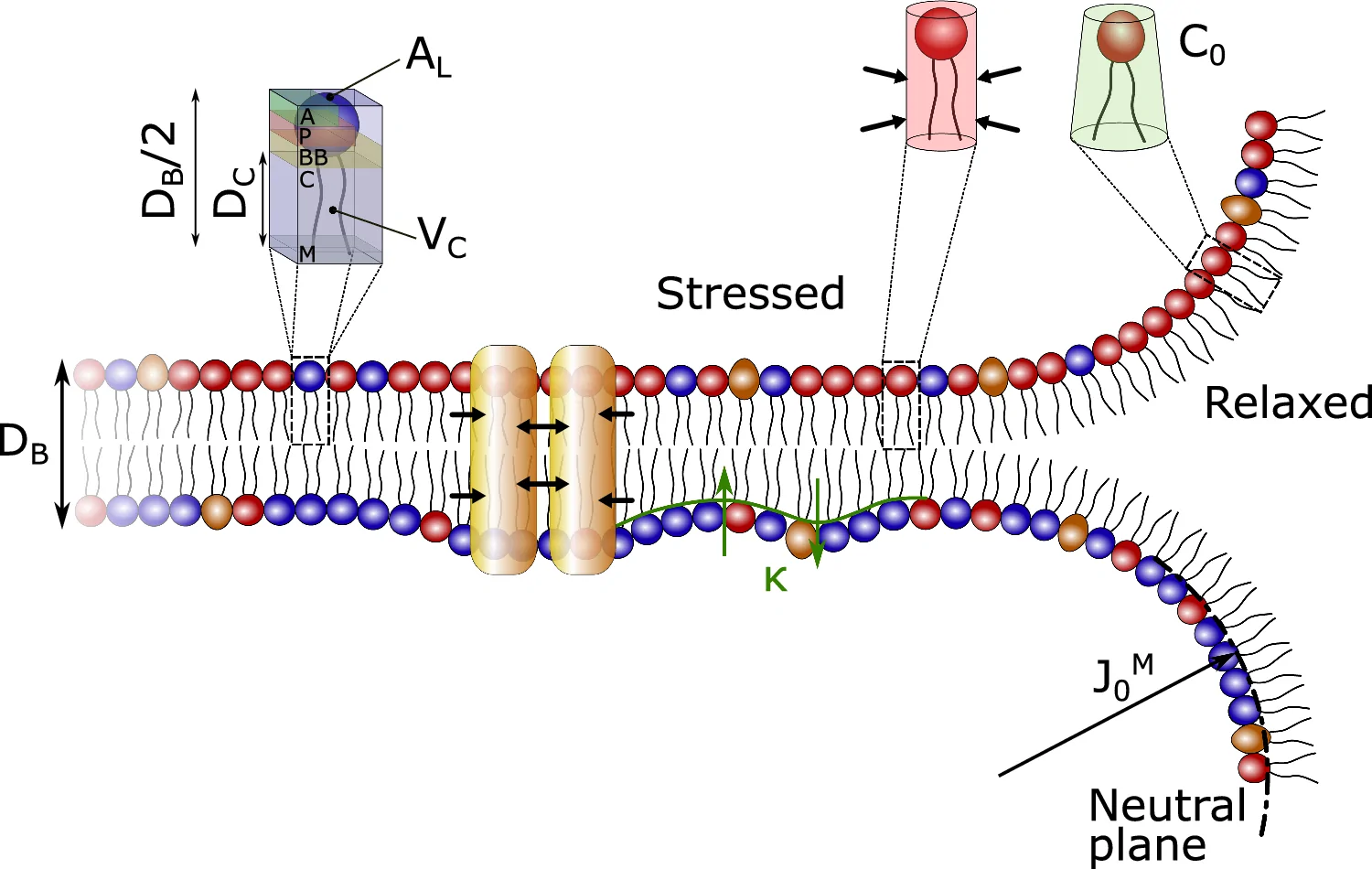
Schematic representation of membrane structural properties assessable by small-angle scattering techniques. The membrane structure is described through volume distribution functions (Sect. 2.1), exemplified here for hydrocarbon chains (C), their termini (M), the lipid backbone (BB), the phosphate group (P), and the alcohol (A) attached to the phosphate. These volume distribution functions define key bilayer properties, including bilayer thickness ($D_B$), hydrocarbon chain thickness ($D_C$), hydrocarbon chain volume ($V_C$), and the area per lipid ($A_L$). The depicted lipid bilayer features an asymmetric distribution of phospholipids across its leaflets, resulting in distinct structural properties for each leaflet (Sect. 2.3). On the right, the schematic illustrates the bilayer split into “virtually” decoupled and relaxed monolayers with a spontaneous curvature ($J_0^M$) derived from the intrinsic curvature (shape) of individual lipids ($C_0$) and their interactions (Sect. 3.1). In bilayers, lipids adopt a stressed state, which, along with membrane bending rigidity (κ) (Sect. 3.2), may influence the function and interactions of integral membrane proteins (Sect. 3.3)

Building on the pioneering efforts of Vittorio Luzzati’s laboratory to characterize diverse lipid mesophases (Tardieu et al. 1973), subsequent work has significantly advanced our understanding of bilayer structure in the biologically most relevant fluid lamellar phase (Wiener and White 1991; Nagle and Tristram-Nagle 2000). Today, leveraging the distinct structural contrasts provided by SAXS and SANS experiments, combined with advanced optimization algorithms, enables detailed insights into molecular packing and lipid structures. These approaches can characterize coexisting domains or asymmetric bilayers without requiring bulky labels. Figure 1 summarizes the structural and elastic parameters of membranes that are accessible through small-angle scattering experiments, as explored in this article.

Retrieving detailed membrane structure from scattering data requires supplementary information, such as lipid volumes or molecular group volumes (Nagle et al. 2019), to constrain the analysis. These methods, commonly referred to as ’compositional modeling,’ have been comprehensively reviewed in recent literature (Semeraro et al. 2021, 2023). In this review, we focus instead on showcasing diverse applications of small-angle scattering on lipid membranes, as illustrated in Fig. 1, with an emphasis on results from our laboratory. Additionally, we discuss our recent contributions to scattering studies of live cells, including applications related to antimicrobial peptides (Sect. 4).

## Membrane structure: from single component bilayers to complex lipid mixtures

### Homogeneous lipid membranes

X-rays and neutrons can probe the structure of lipid membranes in two primary configurations: aligned membranes, for example, tethered to a solid substrate, or free-floating vesicles. The choice of membrane topology is highly dependent on the specific research question. This review focuses exclusively on lipid vesicles, which can be prepared as either multilamellar vesicles (MLVs) or unilamellar vesicles of defined size—typically around 100 nm, commonly referred to as large unilamellar vesicles (LUVs). The membrane structural details retrievable from these vesicles do not depend on whether LUVs or MLVs are studied; however, MLVs provide additional information about the balance of attractive and repulsive forces between membranes. By analyzing the shape of Bragg peaks in SAXS patterns of MLVs, researchers can extract the fluctuation (or Caillé) parameter (Caillé 1972), which is linked to the bilayer bending rigidity and the bulk inter-membrane interaction modulus (Pabst et al. 2000). SAXS experiments hold an advantage over SANS in this context due to the significantly lower wavelength spread ($\Delta \lambda / \lambda$) of X-rays compared to neutrons, enabling more precise characterization of the Bragg peak shape. Additionally, osmotic stress methods, such as the addition of large neutral polymers, allow for probing inter-membrane forces and measuring bilayer bending rigidity (Petrache et al. 1998; Lu et al. 2016). Section 2.2 provides an example of such measurements.

**Fig. 2:**
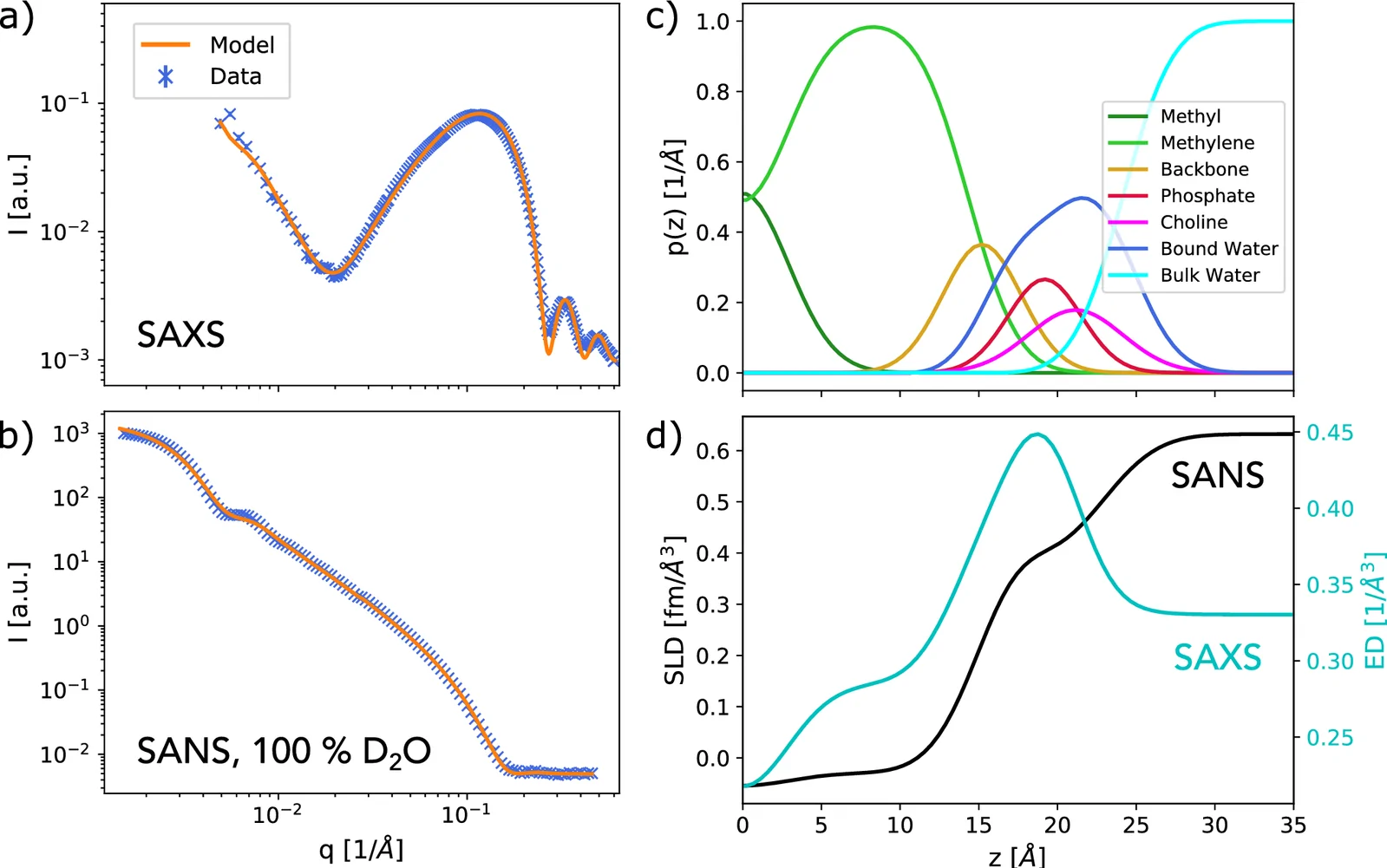
SAXS (**a**) and SANS (**b**) data of DPPC at 50$^\circ$C with the respective model fits using an SDP model. (**c**) The volume probability functions describing the bilayer cross section, including those of bulk and bound water. Note that the density of bound water is about 3% higher than in bulk. The resulting neutron SLD (black) and electron density profiles (cyan) are drawn in (**d**). Figure adapted from Frewein et al. (2021) with permission of MDPI (CC-BY 4.0 DEED license)

The structure of lipid membranes can be described using various models, each with differing levels of complexity and information content (Semeraro et al. 2023). Among these, the scattering density profile (SDP) model (Klauda et al. 2006) has proven particularly effective, enabling the joint analysis of SAXS and SANS data for both LUVs (Kučerka et al. 2009) and MLVs (Heftberger et al. 2014). In essence, the SDP model represents the time-averaged positional fluctuations of lipid molecules and their moieties using Gaussian-type distribution functions that describe lipid headgroups, backbones, and acyl tails as quasimolecular groups. The specific parsing of these groups depends on the research question and the available scattering contrast. This contrast, known as the excess scattering length density (SLD), is determined by the atomic composition and volume of the quasimolecular group relative to the SLD of the aqueous solution. SANS experiments can exploit hydrogen/deuterium exchange to vary the contrast (Semeraro et al. 2021, 2023), which, when combined with SAXS data, enables comprehensive analyses tailored to the research objective. Figure 2 illustrates a joint SAXS/SANS analysis of DPPC LUVs, which revealed a bound water layer in the lipid headgroup region with a density distinct from bulk water (Frewein et al. 2021).

**Fig. 3:**
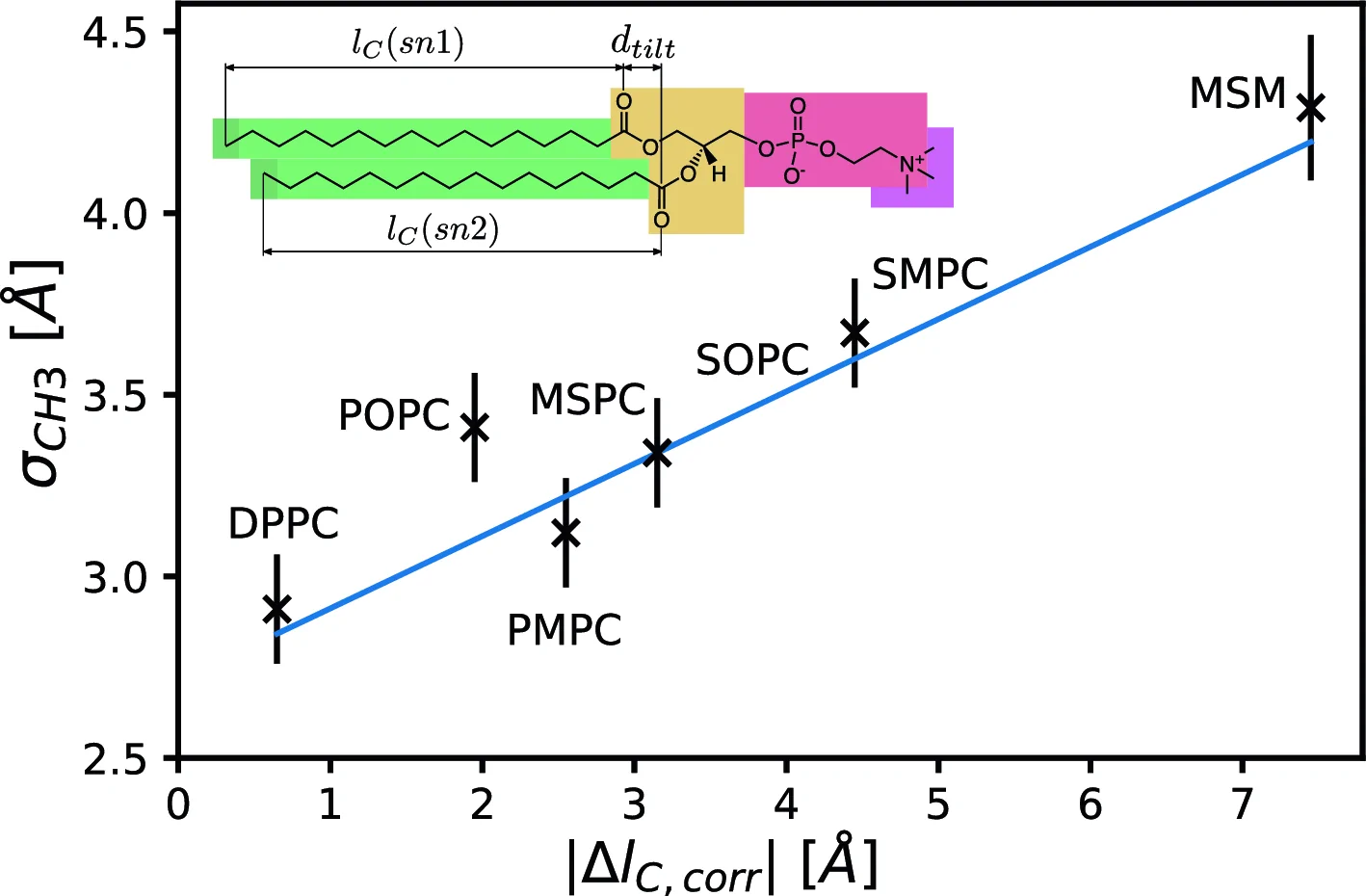
Width of the Gaussian function describing the methyl terminus, $\sigma _\text {CH3}$, as a function of the hydrocarbon chain length difference, corrected by the tilt of the lipid backbone, $\Delta l_\text {C,corr}$ for lipids of varying hydrocarbon mismatch. The solid line shows a linear fit that leads to the determination of $d_\text {tilt}= 0.48\,\mathrm{\AA}$. Figure adapted from Frewein et al. (2021) with permission of MDPI (CC-BY 4.0 DEED license)

Compositional modeling has also been applied to study the hydrocarbon chain overlap (interdigitation) as a function of chain length asymmetry (Frewein et al. 2021). This overlap is associated with the width of the Gaussian distribution describing the terminal methyl group ($\sigma _{\text {CH3}}$). Incorporating the contribution of lipid backbone tilting ($d_{\text {tilt}}$), which describes deviations from a flat orientation relative to the membrane surface, enabled the derivation of average tilt values for a series of phosphatidylcholine lipids and MSM (Fig. 3). Lipid backbone tilting is well-documented for lamellar gel phases (Nagle et al. 2019). Our findings for the fluid phase indicate an average tilt of $d_{\text {tilt}} = 0.48$Å, approximately half the length of a CH$_2$ segment (Frewein et al. 2021), and about four times less than in the gel phase.

### Lipid domains

Ternary and quaternary mixtures of low-melting phospholipids, high-melting phospholipids, and cholesterol are well known for exhibiting coexisting liquid-ordered (Lo) and liquid-disordered (Ld) domains across various compositions and temperatures (Ackerman and Feigenson 2015). While numerous experimental techniques have investigated the size and composition of Lo/Ld domains, SANS combined with appropriate contrast variation schemes enables the measurement of domain sizes on the scale of approximately 10 nm (Heberle et al. 2013), well below the resolution limit of optical microscopy. Interestingly, like domains can stack atop one another in MLVs, an effect that SAXS experiments can exploit to determine the thickness and area per lipid of coexisting Lo/Ld domains (Heftberger et al. 2015; Belička et al. 2017). Lo domains are not only thicker than Ld domains, but are also more densely packed with lipids. These distinctions in thickness and lateral area per lipid between Lo and Ld domains diminish as domain size decreases from a few micrometers to several nanometers (Belička et al. 2017).

Interactions between like domains have also been investigated using osmotic stress experiments (Kollmitzer et al. 2015). These studies revealed that Ld domains experience stronger repulsive forces than Lo domains, primarily due to increased hydration forces and bending fluctuations. This behavior is likely attributable to the lower lipid packing density within Ld domains.

### Membrane asymmetry

Plasma membranes of both prokaryotes and eukaryotes exhibit an asymmetric transbilayer distribution of lipids (Boon and Smith 2002; Lorent et al. 2020). The biological necessity of maintaining this asymmetry, however, remains unclear. Recently, we proposed that the enrichment of saturated phospholipids in the exoplasmic leaflet and (poly)unsaturated phospholipids in the cytoplasmic leaflet of eukaryotic membranes may enable cells to balance the barrier function of the membrane with sufficient fluidity for intracellular signaling (Schütz and Pabst 2023). The development of methods for fabricating asymmetric lipid vesicles (Krompers and Heerklotz 2023) presents an exciting opportunity to investigate this and related questions in detail.

Using 100 nm-sized asymmetric large unilamellar vesicles (aLUVs) produced via cyclodextrin-mediated lipid exchange (Doktorova et al. 2018), we developed analytical tools to characterize the structure and composition of each membrane leaflet (Eicher et al. 2017). These tools leverage the distinct neutron scattering contrast between hydrogen and deuterium by incorporating phospholipids with deuterated hydrocarbon chains in addition to protiated phospholipids alongside D$_2$O/H$_2$O solvent mixtures. This approach allowed us to examine the extent of structural coupling between opposing membrane leaflets.

**Fig. 4:**
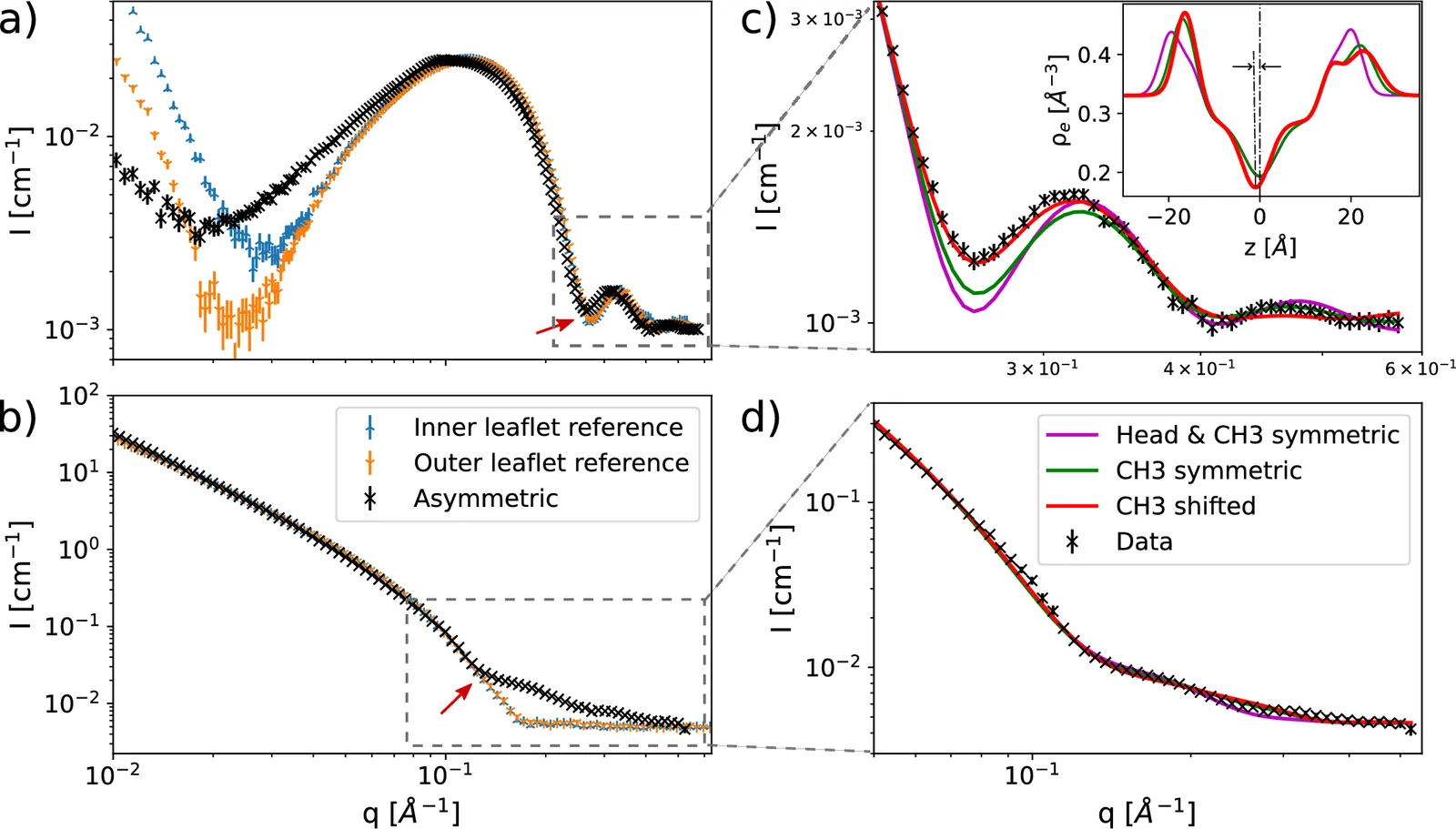
Joint SAXS/SANS analysis of aLUVs composed of a chain-deuterated DPPC-enriched inner leaflet and an SMPC/DPPC (70:30 mol/mol) mixture in the outer leaflet. (**a**) Compares the SAXS data of the aLUVs (black symbols) with the scattering from the corresponding inner (blue symbols) and outer leaflet mixtures (orange symbols). (**b**) SANS data of the same vesicles. Panels (**c**) and (**d**) highlight the analysis of aLUVs in the *q*-range where the scattered intensity shows a lift-off when compared to that of symmetric vesicles, indicated by dashed boxes in panels (**a**) and (**b**). In this *q*-range, SANS is sensitive to the asymmetric distribution of deuterated and protiated hydrocarbons and SAXS to the interdigitation of hydrocarbons. The latter effect leads to a shift of the position of the methyl trough away from the center of the bilayer (see insert in **c**). Figure adapted from Frewein et al. (2022) with permission (CC-BY 4.0 DEED license)

Our initial studies demonstrated that a fluid lipid leaflet reduces the packing density of an opposing leaflet in the gel phase, rendering it more fluid-like (Heberle et al. 2016; Eicher et al. 2018). Detecting structural differences between the leaflets in the fluid phase required advancing the SDP modeling, particularly including hydrocarbon chain interdigitation (Frewein et al. 2022). These principles are illustrated in Fig. 4. Asymmetrically distributing deuterated and protiated phospholipids across the membrane, leads to a distinct lift-off in scattered intensity (Fig. 4b), providing direct (model-free) evidence of lipid asymmetry. This intensity lift-off is also found in the SAXS pattern and was attributed to a displacement of the methyl termini centers of mass ($z_\text {CH3}$) due to asymmetric chain lengths. Alternative models with centered methyl termini and symmetric headgroup structures failed to reproduce the experimental data (Fig. 4c).

Structural parameters for aLUVs at 50 $^\circ$C with varying chain-asymmetric lipids in the outer leaflet (and inner leaflets enriched in DPPC) are summarized in Table 1. Compared to symmetric LUVs composed of equivalent lipid mixtures, the bilayer thickness ($D_B$) of aLUVs was generally reduced, except for MSM/DPPC systems. This reduction in $D_B$ is partially attributed to hydrocarbon chain interdigitation into the inner leaflet, as indicated by negative $z_\text {CH3}$ values. Detailed analysis of each leaflet revealed additional insights: the inner leaflet was slightly thinner on average than the outer leaflet, and the area per lipid in the inner leaflet ($A_L^\text {in}$) was consistently larger than in symmetric reference samples. Notably, $A_L^\text {in}$ decreased when MSM dominated the outer leaflet composition. MSM, a natural lipid mixture with long acyl chains (e.g., 22:0, 23:0, 24:0, 24:1), introduces a pronounced length mismatch in hydrocarbon chains. Thus, long MSM’s acyl chains interdigitate deeply into the inner leaflet, inducing lateral bilayer condensation. Conversely, when MSPC, SMPC, or PMPC dominate the outer leaflet, minor acyl chain interdigitation into the inner leaflet apparently disrupts hydrocarbon chain packing leading to an increase of $A_L^\text {in}$.

**Table 1:** Leaflet specific structure of aLUVs with outer leaflets enriched in different chain asymmetric phosphatidylcholines and inner leaflets almost entirely composed of DPPC

Parameter	MSPC	SMPC	PMPC	MSM
$D_B$ [Å]	37.0 (39.6)	38.1 (39.5)	36.1 (38.6)	40.9 (40.0)
$z_\text {CH3}$ [Å]	- 0.96	- 0.95	- 0.69	- 2.59
$D_C^\text {in}$ [Å]	13.2 (14.5)	13.5 (14.6)	13.2 (14.1)	14.8 (14.3)
$D_C^\text {out}$ [Å]	14.0 (14.5)	14.5 (14.4)	13.2 (14.0)	16.4 (16.1)
$A_L^\text {in}$ [Å$^2$]	67.4 (62.4)	65.8 (62.1)	67.5 (63.7)	60.9 (64.2)
$A_L^\text {out}$ [Å$^2$]	65.8 (62.2)	63.5 (62.7)	66.5 (61.7)	63.9 (63.2)

The outer leaflet lipid exchange varied between ∼50% for MSPC and ∼70% for all other lipids. The brackets show the structural results for symmetric membranes composed entirely of inner and outer leaflet lipids. Data taken from Frewein et al. (2022)

The observed changes in $A_L^\text {in}$ in response to outer leaflet composition provide compelling evidence of hydrocarbon chain interdigitation-mediated structural coupling in asymmetric membranes, most likely by balancing configurational entropy of the interdigitating acyl chain, with an optimization of hydrocarbon cohesive forces. A second coupling effect emerged when outer leaflets were enriched in POPC and SOPC. These lipids induced a similar disordering effect of the inner leaflet despite showing only negligible interdigitation. Instead, the large $A_L$ mismatch between DPPC and SOPC/POPC was implied to disorder the inner leaflet (Frewein et al. 2022).

Finally, the lift-off in SANS experiments (Fig. 4b) also provides a means to probe passive lipid flip-flop (Marx et al. 2021). Complementary H-NMR experiments, combined with shift reagents, have been used to assess the stability of membrane asymmetry. Our previous findings revealed lipid flip-flop half-lives of approximately 6 days in the fluid phase (Marquardt et al. 2017).

## Membrane curvature

### Intrinsic lipid curvatures

Lipids are compelled to adopt cuboid or cylindrical shapes in flat membranes, forming the foundation for the development of the SDP model to describe membrane structure (Fig. 1). In the hypothetical scenario of completely decoupled membrane leaflets, each monolayer can curve away from the planar geometry, adopting what is referred to as the spontaneous monolayer curvature, $J_0^M$. Each lipid species contributes to $J_0^M$ through its effective shape, determined by its physicochemical properties and classified by its intrinsic lipid curvature, $C_0$. $C_0$ is defined at the neutral plane where molecular bending and lateral stretching modes are decoupled (Kozlov 2007).

Lipids with an inverted conical shape, such as PEs, induce monolayers that curve toward the aqueous phase, defined as negative intrinsic curvature ($C_0 < 0$). Conversely, cone-shaped lipids (e.g., lyso-lipids) form monolayers that curve away from the aqueous phase ($C_0 > 0$), while cylindrical lipids ($C_0 = 0$) produce flat monolayers (Shearman et al. 2006). Deriving $J_0^M$ from the $C_0$ values of its constituent lipids is highly non-trivial. Significant differences in lipid shapes can lead to non-linear curvature effects (Kaltenegger et al. 2021), and specific lipid–lipid interactions, such as intermolecular hydrogen bonding, yield non-additive contributions (Sodt et al. 2016). Consequently, $J_0^M \ne \langle C_0^i \rangle$, where the right-hand term represents the compositional average of all $C_0$ values.

The inverted hexagonal phase (H$_\text {II}$) provides a valuable lipid mesophase for studying $C_0$ using SAXS (Alley et al. 2008; Kooijman et al. 2005). In such studies, alkanes or alkenes are often utilized to relieve the stress caused by the need to fill the hydrophobic interstices between individual inverted rod-like micelles. We have employed this approach (Kollmitzer et al. 2013) and further refined SAXS analysis of H$_\text {II}$ phases using a compositional model (Frewein et al. 2019). This methodology allowed us to characterize the intrinsic curvatures of DOPE, POPE, and DMPE. DOPE forms an H$_\text {II}$ phase over a broad temperature range, making it an ideal template for determining $C_0$ values of lipids that do not form such phases. However, this approach is complicated by the aforementioned non-linear and non-additive mixing effects. By accounting for non-linear contributions arising from significant headgroup size differences between DOPE and various guest lipids, we derived $C_0$ values for a range of phospholipids and sphingolipids (Kaltenegger et al. 2021). These results are summarized in Table 2.

The data in Table 2 were predominantly calculated assuming ideal lipid mixing. Our studies, however, reveal deviations from this ideal behavior for sphingolipids, emphasizing the influence of hydrogen bonds that contribute to non-additive mixing effects (Sodt et al. 2016). Table 2 also includes recent data for POPG (Piller et al. 2024). In pure water, POPG exhibits a slightly positive $C_0$. In the presence of calcium ions, this shifts to significantly negative values due to strong divalent ion binding to up to two lipid headgroups (Yesylevskyy et al. 2023). A similar effect is observed with sodium ions, albeit requiring much higher concentrations. In this case, the ions do not strongly bind, but instead reduce the effective headgroup size of anionic lipids through electrostatic screening (Lu et al. 2016).

**Table 2:** Collection of $C_0$ values reported from full *q*-range analysis of H$_\text {II}$ lipid mixtures

lipid	$$C_0$$ (Å$$^{-1})$$	lipid	$$C_0$$ (Å$$^{-1})$$	lipid [ion]	$$C_0$$ (Å$$^{-1})$$
DLPC$^{a,d}$	0.011 (5)	C2:0 Cer$^\textrm{a,d}$	- 0.03 (2)	POPG [–] $^{c,d}$	0.10 (4)
DMPC$^{a,d}$	0.007 (2)	C6:0 Cer$^\textrm{a,d}$	- 0.09 (2)	POPE [Ca$^{2+}$] $^\textrm{c,d}$	- 0.0331 (3)
DPPC$^\textrm{a,d}$	0.005 (5)	C16:0 Cer$^\textrm{a,d}$	- 0.11 (2)	POPG [Ca$^{2+}$] $^\textrm{c,d}$	- 0.020 (2)
DSPC$^\textrm{a,d}$	0.008 (3)	C24:0 Cer$^\textrm{a,d}$	- 0.15 (3)	POPE [Na$^{+}$] $^\textrm{c,d}$	- 0.0333 (3)
DOPC$^{a,d}$	-0.004 (4)	DOPE$^{a,d}$	-0.041 (1)	POPG [Na$^{+}$] $^{c,d}$	-0.015 (2)
POPC$^\textrm{a,d}$	0.001 (4)	POPE$^\textrm{b,d}$	- 0.0317 (7)		
DOPhPC$^\textrm{a,d}$	- 0.021 (1)	C16:1PE$^\textrm{a,d}$	- 0.037 (1)		
pSM$^\textrm{a,e}$	0.010 (6)	DMPE$^\textrm{b,f}$	- 0.0314 (8)		

The table also includes data on the effect of adding 20 mM Ca$^{2+}$ or 200 mM Na$^{+}$ to POPE and POPG. The errors of the last digit are given in brackets

$^\textrm{a}$From Kaltenegger et al. (2021)

$^\textrm{b}$From Frewein et al. (2019)

$^\textrm{c}$From Piller et al. (2024)

$$^\textrm{d}$$35 $$^\circ$$C

$$^\textrm{e}$$50 $$^\circ$$C

$$^\textrm{f}$$80 $$^\circ$$C

### Membrane elasticity

The bending rigidity of membranes, κ, is a critical determinant of cell deformability and plays a significant role in the function of integral membrane proteins through bulk interactions (Marsh 2008). Various experimental techniques are available to measure the bending rigidity of lipid membranes (Dimova 2014; Nagle et al. 2015; Karal et al. 2023). Among these methods are osmotic pressure measurements combined with SAXS on MLVs, as previously discussed. Additionally, inelastic neutron scattering, particularly neutron spin echo (NSE) spectroscopy, provides a powerful tool for probing membrane bending rigidity (Nagao and Seto 2023).

**Fig. 5:**
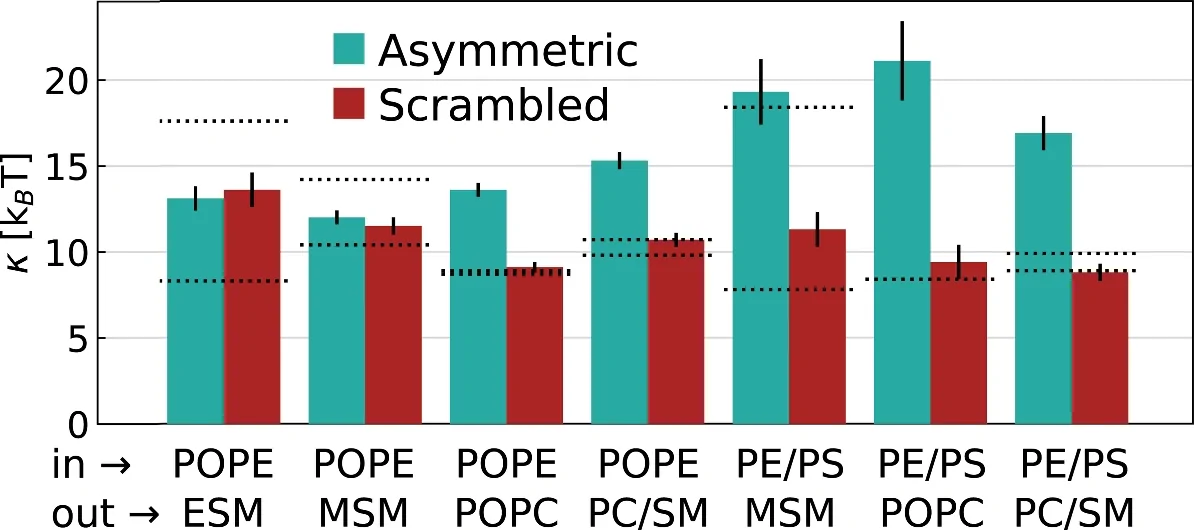
Bending rigidity of asymmetric lipid vesicles (aLUVs) with diverse lipid composition (cyan bars). Data are compared to three symmetric vesicles: (i) scrambled vesicles (red bars), having the same lipid composition as aLUVs, but symmetrically distributed, and (ii) vesicles that entirely composed of either phospholipids of the outer and inner leaflet of the aLUVs (dashed horizontal lines). Note: PE/PS refers to lipid mixture of POPE/POPS (7:3 mol/mol), and PC/SM to POPC/MSM (1:1 mol/mol). Figure taken from Frewein et al. (2023) (CC-BY 4.0 DEED license)

Remarkable findings have been achieved using NSE on asymmetric lipid vesicles (Frewein et al. 2023). The bending rigidity of asymmetric membranes can surpass that of symmetric membranes composed of the same lipids by up to a factor of two (Fig. 5). Analogous results were first reported from asymmetric giant unilamellar vesicle by analyzing the membrane bending fluctuations (Elani et al. 2015). Interestingly, our study showed that some asymmetric membranes exhibit greater rigidity than even their symmetric single-leaflet vesicle counterparts (Frewein et al. 2023). Theoretical models predict that asymmetric membranes may become more rigid due to (i) lateral area differences between leaflets, such as those arising from lipid over- or undercrowding, or (ii) significant differences in $J_0^M$-values between the two leaflets (Hossein and Deserno 2020). However, neither of these scenarios fully explains the observed effects (Frewein et al. 2023). It is hypothesized that intermolecular interactions, such as hydrogen bonding or charge-mediated interactions, may contribute to this unexpected behavior, warranting further experimental investigation.

Despite the incomplete understanding of the underlying mechanisms, these findings unequivocally demonstrate that cell elasticity can be modulated by transitioning between scrambled and asymmetric membrane states using the same lipid composition. Notably, the high elasticity of insect cell membranes correlates strongly with the activity of the scramblase XKR (Shiomi et al. 2021), providing compelling support for this hypothesis.

### Membrane effects on proteins

Membrane protein activity can be modulated by both specific and nonspecific lipid–protein interactions (Corradi et al. 2019). Among the nonspecific interactions, two phenomena have been extensively studied: the hydrophobic mismatch between membrane thickness and the length of the protein’s transmembrane domain (Killian 1998) and curvature stress-related effects (Dan et al. 1994; Cantor 1997). The latter can be characterized by membrane bulk properties, such as $J_0^M$, κ, or $D_B$, which are accessible via elastic scattering experiments (Fig. 1). For instance, we previously demonstrated that the partitioning of integral membrane proteins into liquid-ordered (Lo) or liquid-disordered (Ld) domains is influenced by bulk curvature stress and the geometric shape of the protein (Frewein et al. 2016).

**Fig. 6:**
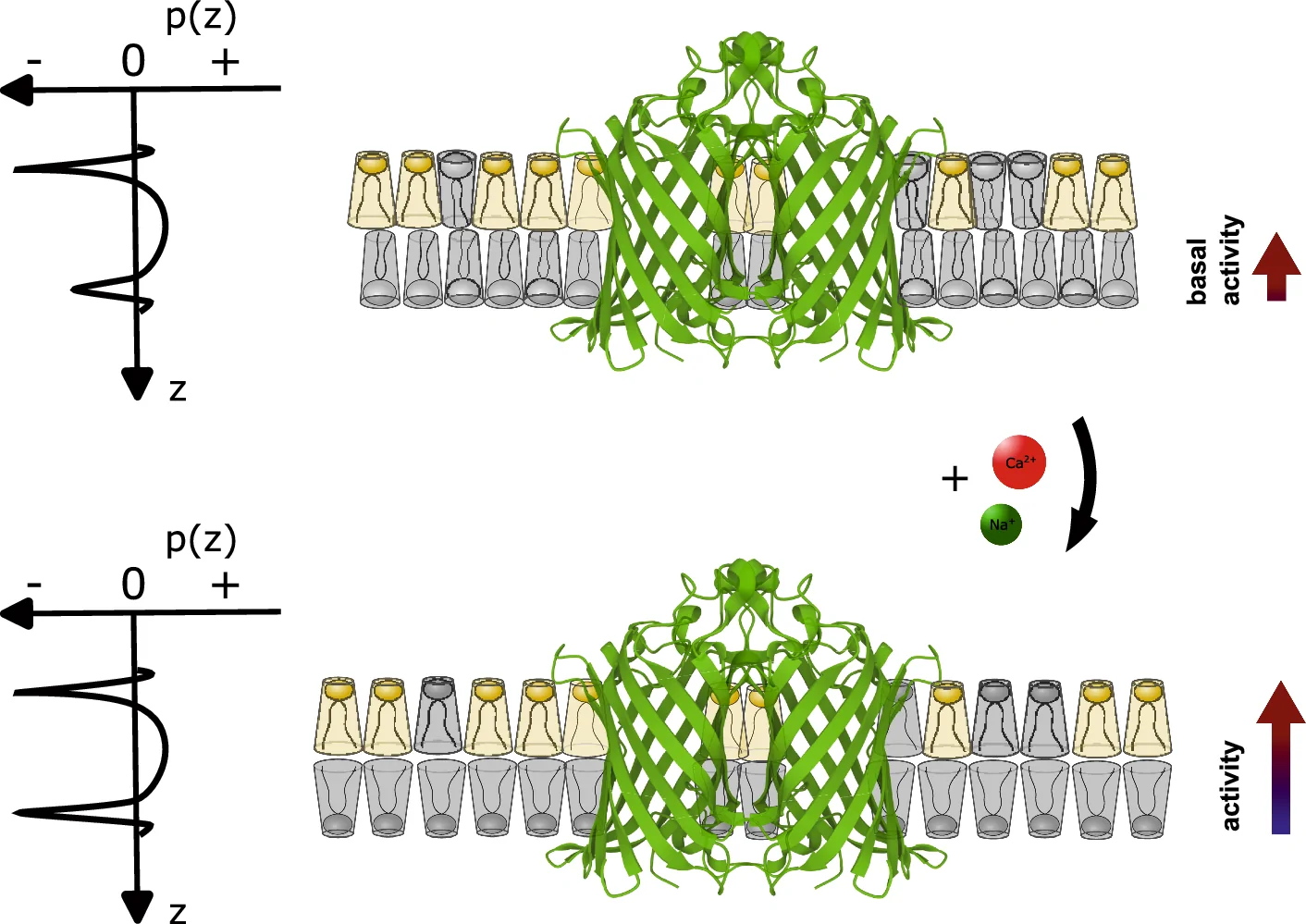
Schematic of coupling between the ion-mediated change of POPG shape (gray lipid) in an asymmetric membrane containing an OmpLA dimer (Protein Data Bank code: 1QD6 (Snijder et al. 1999)). The upper scheme depicts the system at basal conditions. POPG has a weakly expressed cone-like shape ($C_0 \gtrsim 0$), while POPE (yellow lipid) adopts an inverted conical shape ($C_0 < 0$). Imposing a packing into flat monolayer creates significant curvature stress in the POPE enriched leaflet, but not in the POPG-rich leaflet. This leads to differential curvature stress between the two leaflets as indicated by the asymmetric lateral pressure profile *p*(*z*). With POPG adopting a more inverted cone-like shape in the presence of Ca$^{2+}$ or Na$^+$ (lower panel) the curvature stress in the POPG-rich leaflet also increases. However, the differential lateral stress between the membrane leaflets is now partially alleviated (*p*(*z*) becomes more symmetric), leading to an increase of enzyme activity. Figure taken from Piller et al. (2024) (CC-BY 4.0 DEED license)

A particularly intriguing area of research is the coupling of protein activity to membrane asymmetry. Notably, the asymmetry of plasma membranes correlates with the asymmetric shapes of integral membrane proteins (Lorent et al. 2020). Several studies suggest that integral membrane proteins leverage membrane asymmetry to support their specific functions (Pabst and Keller 2024).

Recently, we investigated the activity of the outer membrane phospholipase A (OmpLA) in symmetric and asymmetric lipid membranes. In charge-neutral membranes composed of POPE and POPC, we observed up to a two-order-of-magnitude reduction in the enzymatic activity of OmpLA in asymmetric bilayers compared to symmetric bilayers containing the same lipids (Piller et al. 2023). This behavior can be quantitatively explained using a simple allosteric model that incorporates differential curvature stress in asymmetric bilayers, largely driven by the differing intrinsic curvatures of POPE and POPC (Table 2).

The influence of intrinsic lipid curvature on OmpLA activity becomes even more pronounced in lipid mixtures containing POPG. Calcium, a known cofactor that stabilizes OmpLA in its active dimer form (Dekker 2000), also shifts the $C_0$-value of POPG toward more negative values through interactions with its headgroups (Table 2). This change makes the molecular shape of POPG more similar to POPE, which does not significantly alter its shape in the presence of Ca$^{2+}$ (Table 2). Similar shifts in the $C_0$-value of POPG can also be induced by Na$^{+}$, albeit at much higher concentrations. However, Na$^{+}$ is not a cofactor for OmpLA activity, allowing us to test whether ion-induced differential stress alone is sufficient to activate the protein. Remarkably, OmpLA achieved lipid hydrolysis rates in the presence of Na$^{+}$ comparable to those observed with Ca$^{2+}$ (Piller et al. 2024). Figure 6 illustrates how the curvature stress contribution of POPG changes upon the addition of Na$^{+}$ and Ca$^{2+}$ ions.

Although additional interactions, such as those of electrostatic origin, likely contribute, these examples demonstrate that membrane physical properties accessible through scattering experiments provide profound insights into the complexities of lipid–protein interactions.

## Membrane active compounds: beyond cell wall effects

Membrane-active compounds are a diverse class of molecules that interact with lipid bilayers, modulating their structure and physical barrier function. Among these, significant attention has been devoted to antimicrobial peptides (AMPs), which are amphipathic and typically contain cationic amino acid residues (Wimley and Hristova 2011). The physicochemical properties of AMPs result in diverse effects on membrane structure, including alterations in membrane thickness, deformation, transient pore formation and even complete detergent-like solubilization.

The specific mode of interaction between AMPs and membranes is well known to depend on both the physicochemical properties of the peptide and the composition of the lipid bilayer. However, it is less widely recognized that the partitioning of AMPs into lipid membranes and consequently their effects on membrane structure is highly dependent on the overall lipid concentration (White et al. 1998; Heerklotz and Seelig 2007).

Using a combination of SAXS/SANS experiments, molecular dynamics simulations and peptide partitioning assays, we demonstrated the concentration-dependent effects of the frog peptides PGLa and Magainin 2 on lipid bilayers composed of POPE/POPG mixtures (Pachler et al. 2019; Kabelka et al. 2020, 2022; Semeraro et al. 2022). Our work revealed the peptides’ precise locations within the bilayers, showed their ability to induce lipid curvature (see Sec. 3.1) and to respond to both mean and Gaussian curvature. Partitioning experiments allowed us to connect these effects across multiple techniques, providing a unified perspective. However, understanding the physiological processes involving AMPs in cellular membranes requires delving deeper into complex interactions that extend beyond the simplicity of model lipid bilayers, i.e., whole cells.

Building on earlier work at the ID02 lab of the ESRF, our lab has advanced efforts to develop a refined structural framework for modeling small-angle scattering data from Gram-negative bacteria (Semeraro et al. 2017, 2021). The approach integrates colloidal, polymer and membrane science to create a multiscale analytical scattering model, which simultaneously captures structural details ranging from bacterial ultrastructure (nanoscale) to cell dimensions (submicron scale). It has been rigorously validated using Ultra-SAXS (USAXS), contrast variation SANS and transmission electron microscopy (TEM) with different *E. coli* strains and mutants. This comprehensive approach yielded detailed insights into bacterial architecture, including cytoplasmic and periplasmic metabolite density, lateral packing density of the lipopolysaccharide (LPS) layer, dimensions of the periplasmic space, arrangement of the peptidoglycan layer and whole-cell radius.

**Fig. 7:**
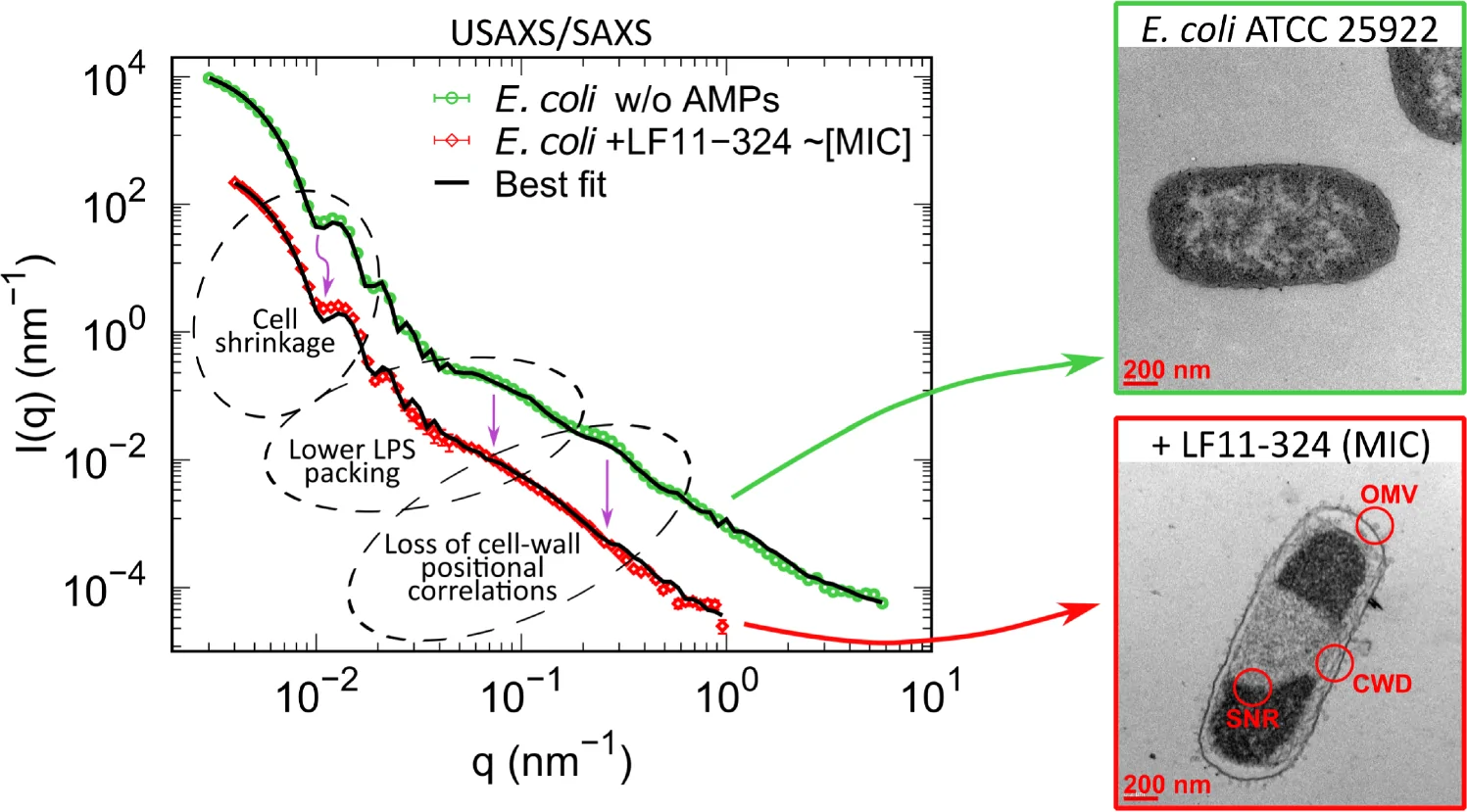
Simplified mapping of USAXS features and direct TEM observations. On the left side, the scattering intensities of *E. coli* cells without (green) and with the application of LF11 peptide (red) at the minimal inhibitory concentration (MIC) are compared. Specific differences such as cell size shrinkage, lowering of LPS package and loss of positional correlations within the cell wall ultrastructure are evidenced at different scattering vector magnitude *q*. The solid lines represent the corresponding fits generated by a multiscale model incorporating parameters such as bacterial size, the spacing between inner and outer membranes, LPS packing density in the outer membrane and the scattering contrast between the cytoplasm and periplasmic space, among others. On the right, TEM images display the formation of outer membrane vesicles (OMV), cell wall disruption (CWD) and separation of the nucleoid region (SNR). Figure adapted from Semeraro et al. (2022) (CC-BY 4.0 DEED license)

The development of this multiscale model allowed us to investigate the structural kinetics caused by lactoferricin-derived (LF11) AMPs (Semeraro et al. 2022). Figure 7 presents a simplified correlation between scattering features and microscopy observations of *E. coli* before and after LF11 treatment. Subsequent stopped-flow USAXS/SAXS experiments revealed key findings, including the kinetics of cytoplasmic and periplasmic leakage, alterations in LPS packing density, cell shrinkage, and the formation of outer membrane vesicles. By combining these insights with AMP partitioning studies on both model membranes and bacterial cells (Marx et al. 2021), we uncovered an unexpected result: the loss of membrane integrity showed only a weak correlation with the antimicrobial efficacy of LF11 peptides. This suggests that interactions between AMPs and cytoplasmic components play a critical role, potentially redefining the principles underlying AMP mechanisms of action.

## Conclusion

Small-angle scattering has long been a cornerstone technique in membrane biophysics, with applications spanning several decades. Complementary to single-molecule approaches, SAXS and SANS offer statistical ensemble averages of membrane structures. Their full potential is realized by harnessing H/D contrast variation in combination with advanced computational optimization techniques. In this review, we highlighted key contributions from the Pabst lab over the past decade. Quantitative analyses of membrane structure and elasticity provide profound insights into mechanisms such as transleaflet coupling in asymmetric membranes, coexisting lipid domains and the general functions of complex lipid mixtures. Notably, these insights shed light on curvature-stress-mediated effects on membrane-active peptides and proteins.

Future research directions aim to further advance these efforts. We anticipate a growing focus on multiscale data modeling, integrating orthogonal information from complementary computational and experimental methods. This intersection of biophysical modeling and experimental validation promises to open new avenues for studying membranes in the context of live cells, likely driving innovative strategies for pharmaceutical development.

## Abbreviations

aLUVs: Asymmetric large unilamellar vesicles
AMPs: Antimicrobial peptides
Cer: Ceramide
DLPC: 1,2-Dilauroyl-*sn*-glycero-3-phosphocholine
DMPC: 1,2-Dimyristoyl-*sn*-glycero-3-phosphocholine
DMPE: 1,2-Dimyristoyl-*sn*-glycero-3-phosphoethanolamine
DOPC: 1,2-Dioleoyl-*sn*-glycero-3-phosphocholine
DOPE: 1,2-Dioleoyl-*sn*-glycero-3-phosphoethanolamine
DOPhPC: 1,2-Diphytanoyl-*sn*-glycero-3-phosphocholine
DPPC: 1,2-Dipalmitoyl-*sn*-glycero-3-phosphocholine
DSPC: 1,2-Distearoyl-*sn*-glycero-3-phosphocholine
Ld: Liquid disordered
Lo: Liquid ordered
LUVs: Large unilamellar vesicles
MLVs: Multilamellar vesicles
MSM: Milk sphingomyelin
MSPC: 1-Myristoyl-2-stearoyl-*sn*-glycero-3-phosphocholine
NSE: Neutron spin echo
OmpLA: Outer membrane phospholipase A
PE: Phosphatidylethanolamine
PMPC: 1-Palmitoyl-2-myristoyl-*sn*-glycero-3-phosphocholine
POPC: 1-Palmitoyl-2-oleoyl-*sn*-glycero-3-phosphocholine
POPE: 1-Palmitoyl-2-oleoyl-*sn*-glycero-3-phosphoethanolamine
POPG: 1-Palmitoyl-2-oleoyl-*sn*-glycero-3-phosphoglycerol
pSM: Palmitoyl sphingomyelin
SANS: Small-angle neutron scattering
SAXS: Small-angle X-ray scattering
SDP: Scattering density profile
SLD: Scattering length density
SMPC: 1-Stearoyl-2-myristoyl-*sn*-glycero-3-phosphocholine
SOPC: 1-Stearoyl-2-oleoyl-*sn*-glycero-3-phosphocholine
